# An Honest Letter to the Nonbelievers in Advanced Pulmonary Embolism Therapy

**DOI:** 10.1016/j.jscai.2022.100569

**Published:** 2023-01-04

**Authors:** Peter Monteleone

**Affiliations:** Department of Medicine, University of Texas at Austin Dell School of Medicine, Ascension Texas Cardiovascular, Austin, Texas

**Keywords:** catheter-directed thrombolysis, mechanical thrombectomy, pulmonary embolism, pulmonary embolism response team

There remain nonbelievers in advanced therapy for pulmonary embolism (PE). We the believers yell at them from podiums and from electronic medical records: “right ventricular (RV) to left ventricular (LV) ratio ≥0.9 is an independent predictive factor for in-hospital mortality!”^1^ or “PE-related mortality at 3 months is 17% if RV/LV ratio is >1.5!”^2^ Sometimes, they yell back “we have treated these patients with anticoagulation alone for decades and they have done *fine*,” to which we smugly respond with a decade of data organized neatly into PowerPoint decks from SEATTLE II^3^ through KNOCOUT PE^4^ and from FLARE^5^ to FLASH^6^ to EXTRACT-PE.^7^ However, if (and when) they respond “there is no guideline recommending these therapies” or they challenge us to “show *any* large randomized data sets recommending taking an intermediate-risk patient to the catheterization laboratory,” the honest among us look down at our blood-covered shoes and kick the catheterization laboratory floor. Then, we look up and look forward to HI-PEITHO^8^ and PE-TRACT,^9^ while we swear honestly that we believe these procedures are the right thing to do for our mutual patients.

In this edition of *JSCAI*, our community continues to probe for weaknesses and layer bricks in the walls of the rapidly growing fortress of advanced PE therapy data. The field has grown exponentially and should be thoroughly commended for the rapidity with which data have been developed and for the sincerity with which dedication has been focused on analysis of real-world and registry data in addition to industry-sponsored results. However, we have not yet achieved large, randomized data and, therefore, have not achieved class 1 therapeutic guidelines. While these large steps are being taken, it remains extremely important to continue to take smaller steps such as those in this edition—continually testing and probing our current practice (which is indeed running ahead of guidelines) and observing where that practice is taking our patients.

Feroze et al^10^ asked whether there were clinical outcomes differences between patients treated with large bore mechanical thrombectomy (MT) versus those treated with catheter-directed thrombolysis (CDT) within their large center with a robust PE response team (PERT). These 2 therapies are so different that answering this question from real-world data is a remarkable challenge. The authors expertly applied proportional hazard regression and propensity matching to perform the fairest possible analysis, but intrinsic differences result in *inherent* bias that really is impossible to overcome. If patients cannot lie down secondary to dyspnea, they may get CDT to allow for jugular venous access. If patients experienced a PE-induced cardiac arrest during an open spine surgery, an operator may favor use of MT over even low-dose thrombolytic with CDT. These variables cannot all be included in regression or propensity analysis, so we are left appreciative for, nonetheless, “poking holes” in, these real-world single-center comparisons.

This same question (MT vs CDT) is being tackled in a prospective, randomized trial sponsored by an industry program that developed one of these therapies in the actively recruiting PEERLESS trial.^11^ PEERLESS will be a large step forward, providing prospective, randomized comparative data, but the PERT community will also ask how fair a comparison of MT by expert MT operators is with CDT by expert MT operators whose programs may have never developed protocolized CDT therapies. We will also, no doubt, reasonably ask whether CDT events in PEERLESS are being adjudicated by an industry sponsor with much to gain from a victory for MT over CDT. Most importantly, regardless of what the study by Feroze et al or PEERLESS trial presents, the *nonbelievers* reading this letter will continue to ask, “regardless of procedural comparisons, should we be doing *any* of this?” The consortia creating guidelines will no doubt continue to ask this same question, but these limitations speak to the value addition of the real-world analytic work performed by Feroze et al: “More bricks in the wall…a continued hunt for cracks and leaks.” We continue to challenge each other to step back and review so that our entire community can move forward.

Bruno et al^12^ probed the available literature for insight into the relative benefits and risks of ultrasound-assisted catheter-directed thrombolysis (US-CDT) versus standard CDT. Their search pooled 7 observational studies and 1 randomized controlled trial and reported no major differences in bleeding events and no differences in pulmonary artery pressure recordings. However, there was a difference in reduction in RV-to-LV ratio favoring standard CDT. The authors did an excellent job reporting the bias risk within their analysis and displaying comparative details of the pooled studies. Regarding bleeding, experienced readers will look at the US-CDT administered thrombolytic dose and note that mean doses for thrombolytic administration were extremely high compared with contemporary CDT. The OPTALYSE PE trial demonstrated the safety and efficacy of 4.0- to 6.0-mg tissue plasminogen activator (tPA) doses.^13^ The KNOCOUT PE registry showed us that between 2018 and 2020 in real-world data, 70.6% of patients treated with the EKOS US-CDT device (Boston Scientific) received <20.0 mg tPA, and 32.4% of patients received <12 mg.^4^ So, what is the value of demonstrating no difference in bleeding rates between standard CDT and US-CDT when the US-CDT arm *mean* dose ranged from 19.0 to 34.4 mg with an SD as high as 17.3? The author of this letter has not given >14.0 mg tPA for CDT since 2019.

Regarding the combined analysis of RV-to-LV ratio reduction, this finding was driven by the work of SUNSET-sPE.^14^ This small but interesting trial was prospectively performed but lacked standardization of thrombolytic dosage. Indeed, in that work, the authors acknowledged that the OPTALYSE PE results affected and altered selection of thrombolytic dosage midtrial in both arms. With widely varied dosages in all studies pooled in this work, it is difficult to definitively compare US-CDT with standard CDT regarding RV-to-LV ratio achievement, especially when many of us believe that the goal of US is not to necessarily affect this outcome *more* but rather to allow us to affect this outcome with progressively lower and thus safer doses of thrombolytic. That does not minimize the authors’ work here. This is the first broad overview of what the literature has given us thus far. It summarizes well, for the first time, everything published regarding what we know and, importantly, what we do not know about this technology. “Another brick in the wall…”

The third related article in this edition is a brick to build a brick. Silver et al^15^ should really be praised for the transparency with which this work presents the process by which a comparative historic cohort was developed and assessed for use in the FLowTriever for Acute Massive Pulmonary Embolism analysis.^16^ Building and studying this cohort is not an easy thing to do, and the authors’ meticulous description of development of this group will be a great value to future critique and acceptance of trials comparing those of this cohort with outcomes achieved with novel devices in the highest risk cohort of patients with PE. The authors pooled 27 studies reviewing 1517 patients, and they acknowledged that they relied on “low-to-moderate-quality evidence” to document mortality, major bleeding, and other complications. Nonetheless, this work is likely the best representation of *reported* outcomes of therapies for high-risk PE available. Now, does this cohort represent outcomes with contemporary *standard-of-care* therapies for patients with high-risk PE? We know that patients were treated in the studied cohorts with effectively every modality of treating PE, but we do not know specifically how often each of these therapies was used. In a world where *standard-of-care* for these patients represents systemic thrombolysis with or without extracorporeal membrane oxygenation and in which publication bias undoubtedly results in nonstandard therapies being preferentially discussed in the literature, the answer to this question is likely “no.” Again, however, a great deal of credit is due to the authors here for transparently presenting how this historic cohort was developed. This is undoubtedly the best summary of the literature’s description of outcomes in patients with high-risk PE and will likely be used repeatedly in the future as a comparator for novel therapeutics. Their work makes future readers aware of cohort limitations and may also interest readers in a third standard-of-care arm for future studies, potentially drawn from PERT Consortium registry data or other real-world analytic data lakes.

So, what is the take-home message in this letter for the nonbelievers? Maybe 5 things: (1) We know that patients with PE and RV dysfunction or enlargement categorized as intermediate-risk by the ESC 2019 criteria *do not* “do fine” with anticoagulation alone, and therefore, we know that we will continue to strive on their behalf to advance and enhance their care.^1^^,^^2^^,^^17^ We swear that we do not do these procedures for relative value unit generation or for Twitter followers but rather because this is how we would want our family members treated if they presented with this syndrome. (2) We know from small industry-sponsored trials and from relatively small published real-world experiences that advanced PE therapies with MT and US-CDT can safely and reliably improve outcomes associated with enhanced survival and morbidity in these patients. We understand that single-arm and historic comparator “trials” such as SEATTLE II,^3^ FLASH,^2^ OPTALYSE,^13^ and APEX-AV^18^ may not *convince* you and that they will not get us to a guideline indication, but these trials helped us to hone and enhance the therapies we use every day. In addition, they helped us to eliminate any role for systemic thrombolytics in these patients, which we already know carry unacceptable risks of major bleeding (number needed to harm, 18) and intracranial hemorrhage (number needed to harm, 78), with no demonstrated long-term benefits.^19^^,^^20^ (3) We know that the risks and the lack of benefits previously associated with archaic therapies for intermediate-risk patients (systemic full- and half-dose thrombolytic CDT, high-dose thrombolytic CDT, and historic attempts at nontailored thrombectomy) are of little use in discussion about implementation of contemporary advanced therapies. Forgive us if we seem annoyed when asked to discuss them with you. (4) This field is progressing faster than nearly any other has in clinical history, and the large randomized prospective data that we all want is in process, led by HI-PEITHO, which is already rapidly enrolling,^8^ and PE-TRACT,^9^ which is set to start enrollment in 2023, to guide us all to thoughtful and precise clinical guidelines for care of such high-risk patients.

Finally (and most relevantly), (5) know that for every minute we spend arguing with you, the nonbelievers, about the patients in front of us in our hospitals, we spend hours, if not days, arguing with each other within the community of believers. The articles presented in this issue of *JSCAI* are great examples of how the discussions within our community continue to expand and grow as we challenge each other to treat both patients in front of us and the generations of patients to follow whose care (or lack thereof) will be dictated by our next steps in the development of this field. So, join us and learn with us, but keep arguing with us because we all know that the future of clinical care is best guided by passionate champions on both sides of every debate.
